# Supplementary material for: IBF-R Regulates IRE1α Post-Translational Modifications and ER Stress in High-Fat Diet-Induced Obese Mice
Source: Nutrients. 2022 Jan 4;14(1):217. doi: 10.3390/nu14010217 (PMC8746979; doi:10.3390/nu14010217)
Supplement: Supplementary file 1 [file nutrients-14-00217-s001.zip › nutrients-1521147-supplementary.pdf]

Supplementary Figure Legends

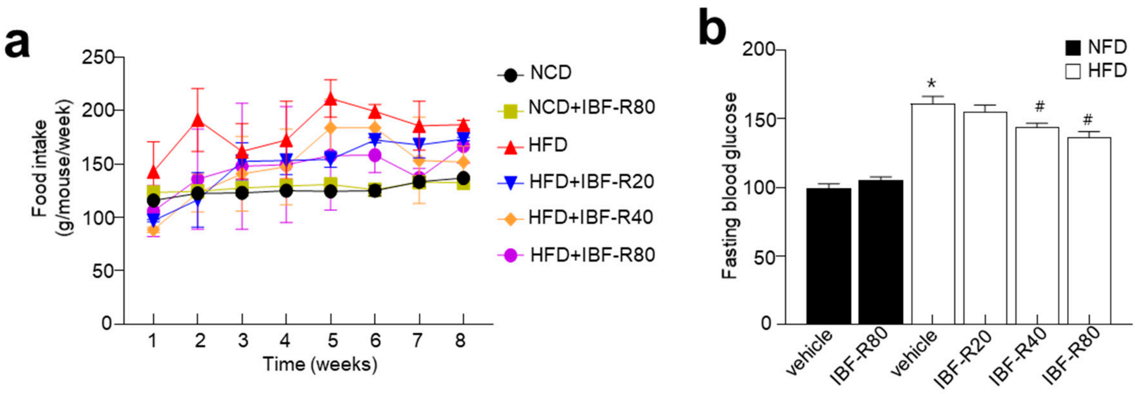

Figure S1. Influence of IBF-R on food intake and blood glucose levels. (a) Quantification food intake during the experimental period. (b) Glucose levels from each group (* $p < 0.05$ vs. NCD + vehicle, # $p < 0.05$ vs. HFD + vehicle).

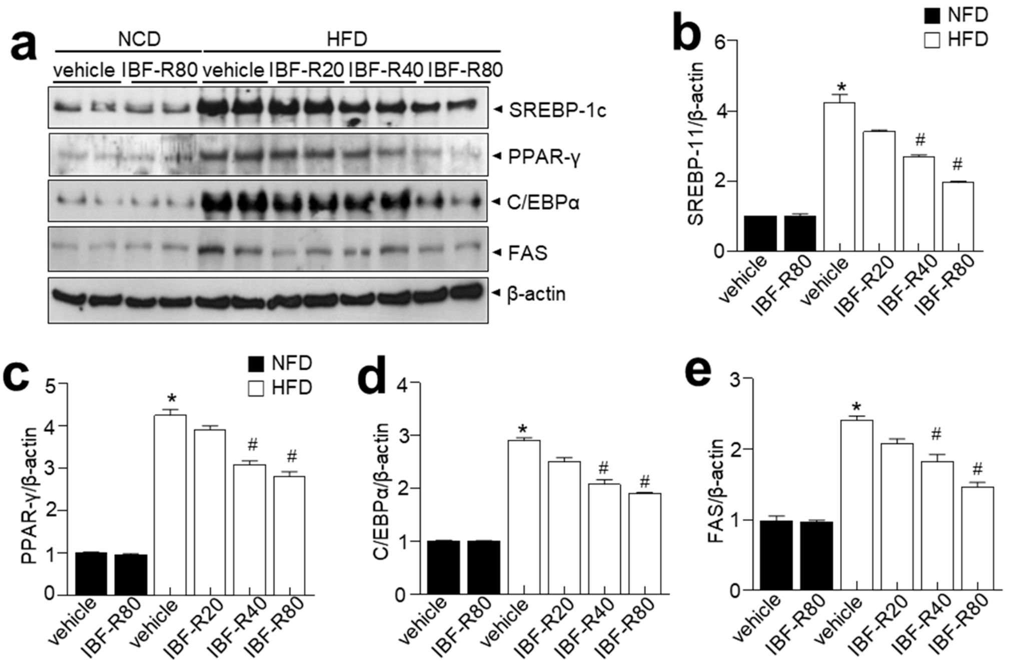

Figure S2. IBF-R determines adipogenic factors in liver tissue. (a) Immunoblotting of SREBP-1c, PPAR-γ, C/EBPα, FAS, and β-actin expressions in liver. (b-e) Quantitative analysis of protein expression was also performed. Data are presented as mean ± SEM (n = 10, * p < 0.05 vs. NCD + vehicle, # p < 0.05 vs. HFD + vehicle). eWAT; epididymal WAT
